# Supplementary material for: Identification of Huge Phages from Wastewater Metagenomes
Source: Viruses. 2023 Nov 28;15(12):2330. doi: 10.3390/v15122330 (PMC10747093; doi:10.3390/v15122330)
Supplement: Supplementary file 1 [file viruses-15-02330-s001.zip › External_supplementary_S1_Supplementary_Figures_and_Tables.pdf]

Supplementary Materials:

Identification of huge phages from wastewater metagenomes

René Kallies [1*], Die Hu [1], Nafi'u Abdulkadir [1], Michael Schloter[2] and Ulisses Nunes da Rocha [1*]

[1] Department for Environmental Microbiology, Helmholtz Centre for Environmental Research, Permoserstr. 15, D-04318 Leipzig, Germany; emails: die.hu@ufz.de; nafiu.abdulkadir@ufz.de

[2] Department of Environmental Health, Helmholtz Munich, Ingolstaedter Landstr. 1 D-85758 Neuherberg, Germany email: schloter@helmholtz-muenchen.de

* Correspondence: ulisses.rocha@ufz.de; rene.kallies@ufz.de

Table of Contents

Figure S1: Viral contig quality 2

Figure S2: Viral contig size distribution 3

Figure S3: Functional annotation categories 4

Figure S4: TBlastX genome-wide comparisons 5

Figure S5: Proteomic subtree of studied phages genomes 6

Table S2. Overview of identified Auxiliary Metabolic Genes. 7

Table S3. Number of transfer RNAs per tRNA type and phage genome 8

Figure S1: Viral contig quality

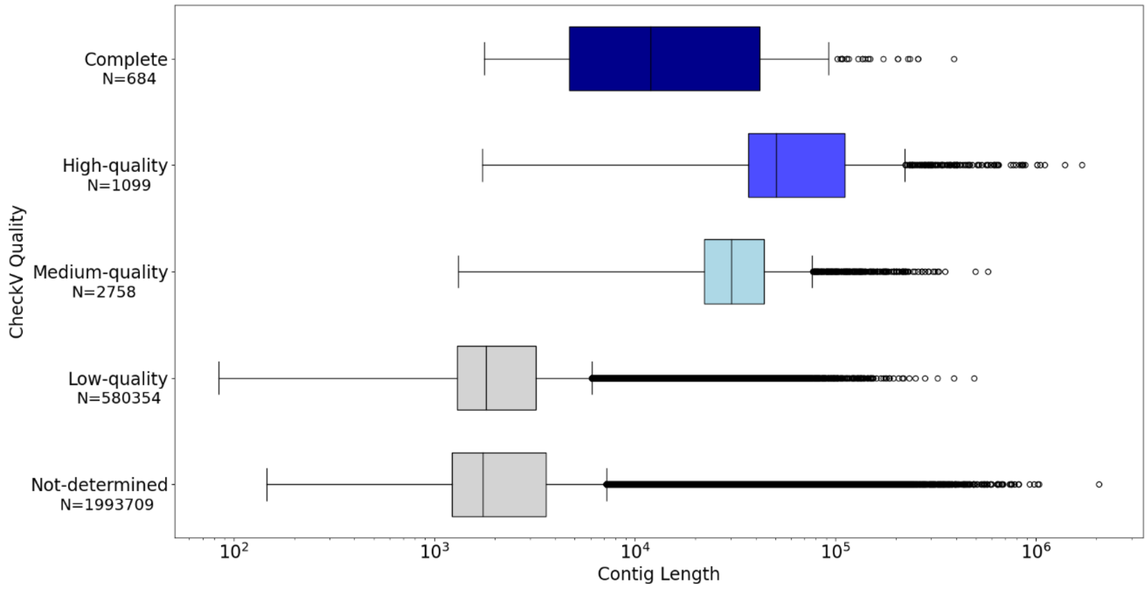

Figure S1. Boxplot presenting the number of metagenome recovered putative viral contigs and their CheckV based quality prediction. Complete (blue) and High-quality viral contigs were used for further analysis.

Figure S2: Viral contig size distribution

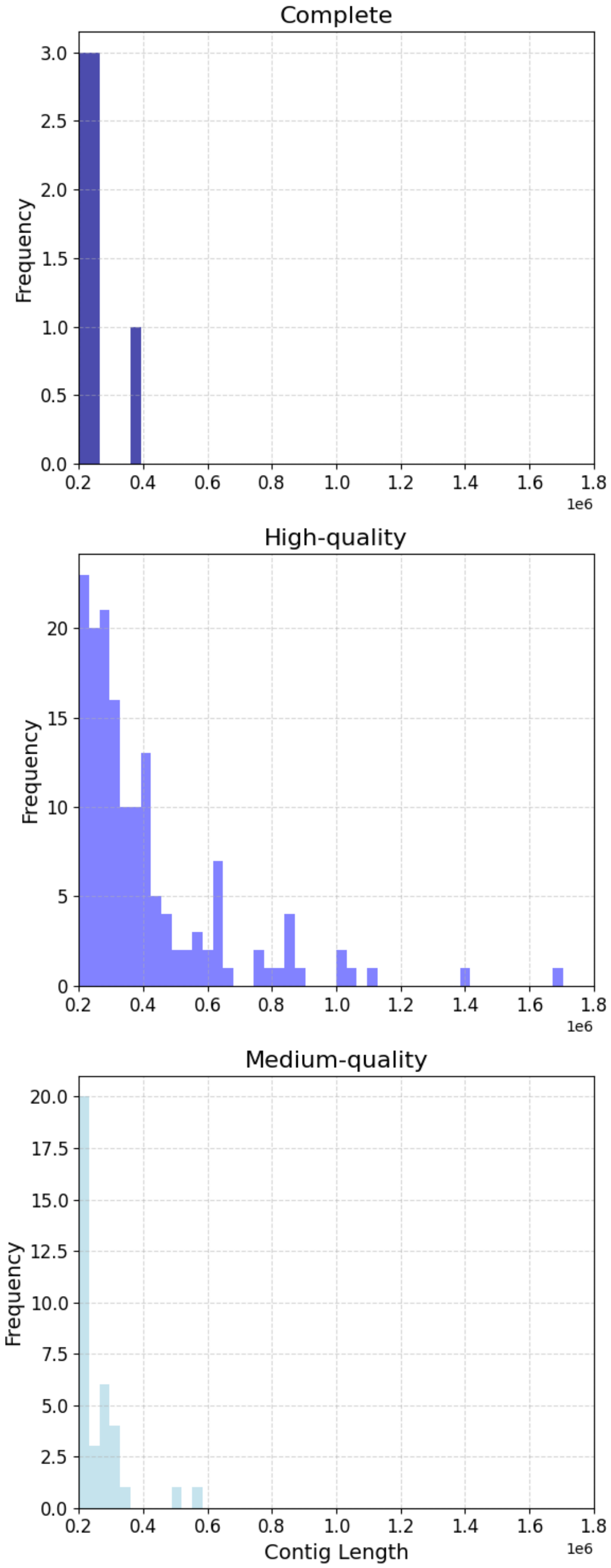

Figure S2. Size distribution for putative viral contigs with a minimum length of 200 kbases (huge phages) shown for CheckV 'Complete' and 'High-quality' categories.

Figure S3: Functional annotation categories

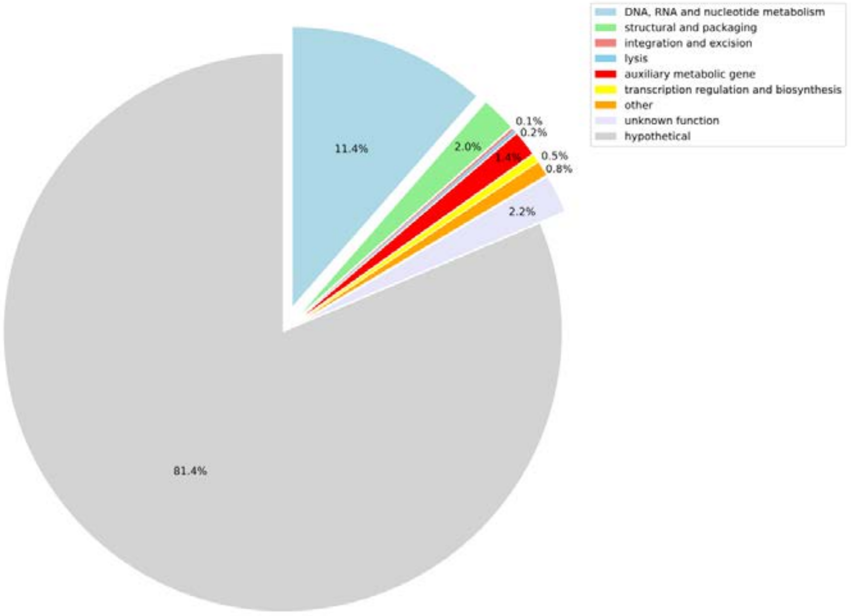

Figure S3. Relative number of functional categories after annotation. A total of 2,861 coding sequences were identified from the seven huge phage genomes.

Figure S4: TBlastX genome-wide comparisons

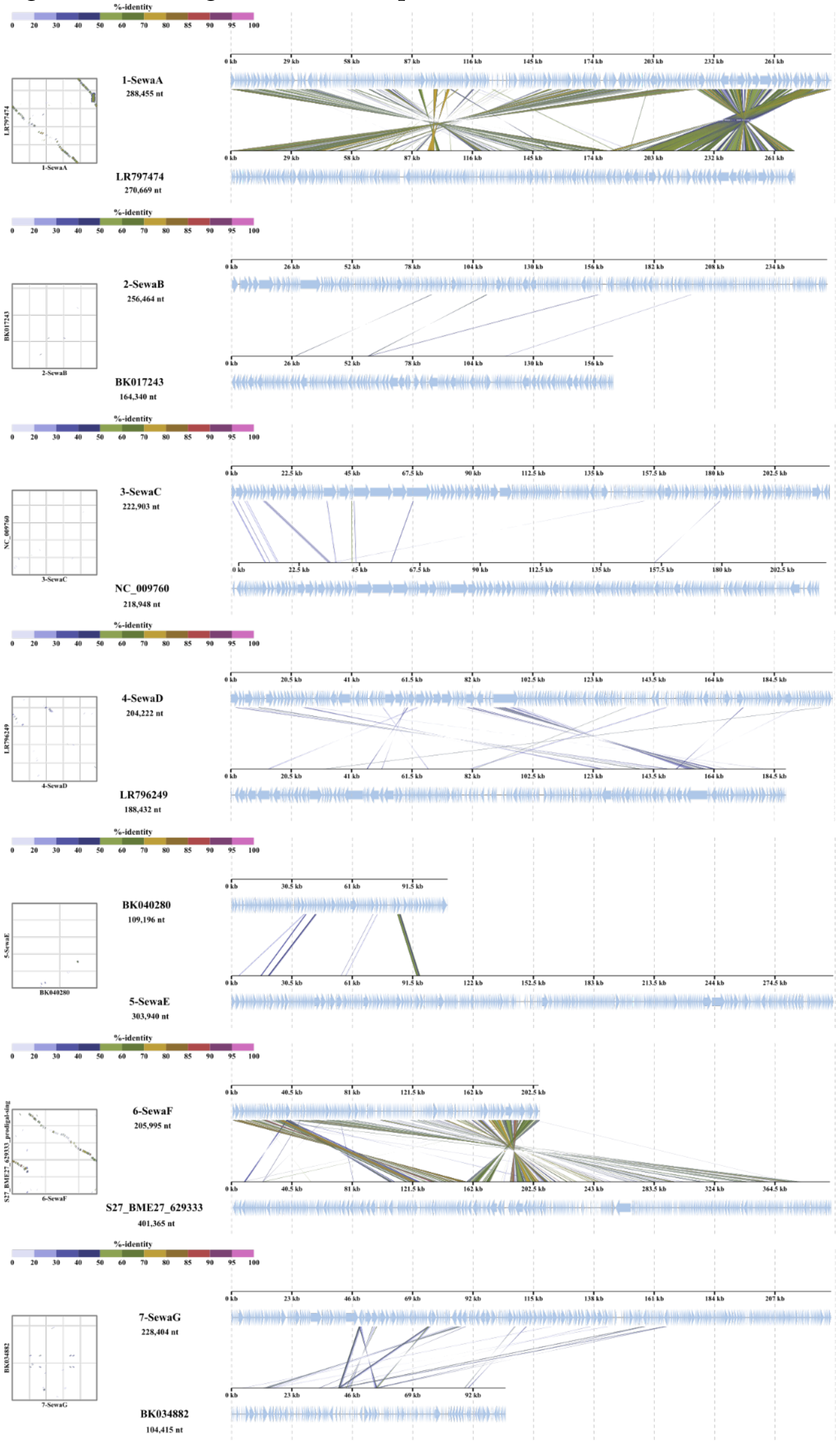

Figure S4. Genome tBlastX comparisons between studied phage genomes and most related phage genomes. LR797474: uncultured Caudovirales phage genome assembly; BK017243: MAG TPA_asm: Caudoviricetes sp. isolate ctmJK21; NC_009760: Bacillus phage 0305phi8-36; LR796249: uncultured Caudovirales phage genome assembly; BK040280: MAG TPA_asm: Bacteriophage sp. isolate ct8zl1; S27_BME27_629333: BM_PHAGE_48_13 – Mahaphage; BK034882: MAG TPA_asm: Caudoviricetes sp. isolate ctDeJ4.

Figure S5: Proteomic subtree of studied phages genomes

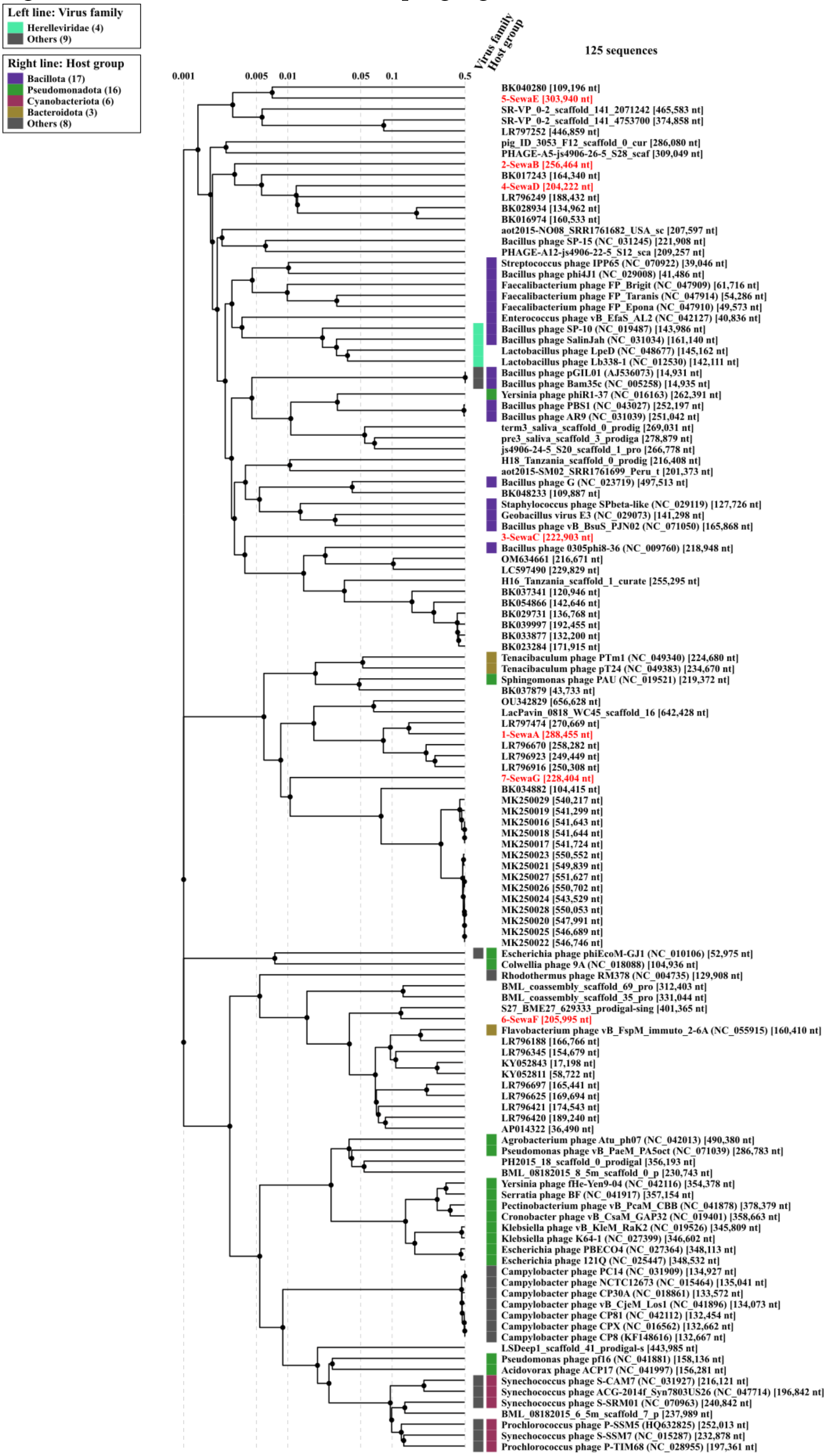

Figure S5. Tblastx-based proteomic subtree of studied phage genomes (shown in red) and their related phage genomes with taxonomic and host information where available. A subtree was selected for the relevant clades.

Table S2. Overview of identified Auxiliary Metabolic Genes.

Contig / Phage	AMG[1] Name	KEGG Ortholog number
1-SewaA	vanY; zinc D-Ala-D-Ala carboxypeptidase	K07260
	purA, ADSS; adenylosuccinate synthase	K01939
	nudB, ntpA; dihydroneopterin triphosphate diphosphatase	K08310
	waaC, rfaC; heptosyltransferase I	K02841
	pigG, redO; peptidyl carrier protein	K21781
2-SewaB	pncA; nicotinamidase/pyrazinamidase	K08281
3-SewaC	TSTA3, fcl; GDP-L-fucose synthase	K02377
	rfbA, rffH; glucose-1-phosphate thymidylyltransferase	K00973
	ahbD; heme synthase	K22227
	rfbD, rmlD; dTDP-4-dehydrorhamnose reductase	K00067
	rfbC, rmlC; dTDP-4-dehydrorhamnose 3,5-epimerase	K01790
	rfbB, rffG; dTDP-glucose 4,6-dehydratase	K01710
4-SewaD	galE, GALE; UDP-glucose 4-epimerase	K01784
	phnP; phosphoribosyl 1,2-cyclic phosphate phosphodiesterase	K06167
	moeB; molybdopterin-synthase adenylyltransferase	K21029
	pncA; nicotinamidase/pyrazinamidase	K08281
	rfbD, rmlD; dTDP-4-dehydrorhamnose reductase	K00067
	wecB; UDP-N-acetylglucosamine 2-epimerase	K01791
	mec; [CysO sulfur-carrier protein]-S-L-cysteine hydrolase	K21140
	pncB, NAPRT1; nicotinate phosphoribosyltransferase	K00763
	rfbC, rmlC; dTDP-4-dehydrorhamnose 3,5-epimerase	K01790
5-SewaE	iscS, NFS1; cysteine desulfurase	K04487
	pncB, NAPRT1; nicotinate phosphoribosyltransferase	K00763
	DNMT3A; DNA (cytosine-5)-methyltransferase 3A	K17398
	araD, ulaF, sgaE, sgbE; L-ribulose-5-phosphate 4-epimerase	K03077
	cobT; cobaltochelatase CobT	K09883
	coaD, kdtB; pantetheine-phosphate adenylyltransferase	K00954
	cobS; cobaltochelatase CobS	K09882
	pncA; nicotinamidase/pyrazinamidase	K08281
	nadD; nicotinate-nucleotide adenylyltransferase	K00969
	phnP; phosphoribosyl 1,2-cyclic phosphate phosphodiesterase	K06167
	PPCDC, coaC; phosphopantothenoylcysteine decarboxylase	K01598
	DNMT1, dcm; DNA (cytosine-5)-methyltransferase 1	K00558
	nadE; NAD+ synthase	K01916
6-SewaF	nadE; NAD+ synthase	K01916
	pncA; nicotinamidase/pyrazinamidase	K08281
	DHFR, folA; dihydrofolate reductase	K00287
	pncB, NAPRT1; nicotinate phosphoribosyltransferase	K00763
7-SewaG	NAMPT; nicotinamide phosphoribosyltransferase	K03462
	queC; 7-cyano-7-deazaguanine synthase	K06920
	K13522, nadM; bifunctional NMN adenylyltransferase/nudix hydrolase	K13522

[1] AMG: Auxiliary Metabolic Gene.

Table S3. Number of transfer RNAs per tRNA type and phage genome

	1-SewaA	2-SewaB	3-SewaC	4-SewaD	5-SewaE	6-SewaF	7-SewaG
**Amino acid**							
standard							
Ala	1	0	0	1	1	2	1
Arg	4	1	1	0	3	5	1
Asn	1	0	0	1	2	1	1
Asp	2	0	0	1	0	1	1
Cys	2	1	0	1	1	1	1
Gln	1	0	0	1	1	2	1
Glu	2	0	0	1	2	1	0
Gly	2	1	0	1	2	2	1
His	1	1	0	1	0	1	0
Ile	2	1	1	3	2	2	2
Leu	5	1	1	2	3	4	3
Lys	3	1	0	2	2	1	1
fMet	2	0	0	0	0	0	0
Met	2	0	0	1	1	3	0
Phe	1	0	0	1	1	0	0
Pro	4	1	1	1	2	3	0
Ser	4	0	0	2	2	3	1
Thr	3	1	0	0	1	3	1
Trp	1	0	0	1	0	0	1
Tyr	1	1	0	1	1	1	0
Val	2	1	0	1	1	2	1
non standard							
Pyl	0	0	0	0	0	0	0
Sec	0	0	0	0	0	0	0
Sup	1	1	1	0	0	0	0
Total	47	12	5	23	28	38	17
Pseudo Genes	2	2	1	2	2	1	3
