# Supplementary material for: Identification of Huge Phages from Wastewater Metagenomes
Source: Viruses. 2023 Nov 28;15(12):2330. doi: 10.3390/v15122330 (PMC10747093; doi:10.3390/v15122330)
Supplement: Supplementary file 1 [file viruses-15-02330-s001.zip › External_supplementary_S4_SI_CRISPR_sequences.pdf]

Supplementary Material: Overview of putative CRISPR sequences

Direct repeats are in shown in yellow and spacers are marked in grey. Blast hits to similar sequences in the Direct Repeats and Spacer databases [1,2] are shown below each sequence (e-value cut-off 0.01).

>5-SewaE-CRISPR_1
TCCGTAGCCTTCTAAGTGAAGCCGCAAGTAATCTCGGAGAGAAATCAAACTTCTCAGAAATC
ATTGCAAAGTATCTTAACCTTAAGGTACAAGAACAACCACAAGCAGGTGGGCAAGAAGGCG
GTGGAGAAGGTGGAATGCCAGACTTGCCTTCTCTATTGGGTGGACAAGCAGGTGGGCAGGAA
GGCGATCAAGAAACCGGTGGACAGGAAGCACAACAAACAAGTGATGAATCTCAGAAATTCC
TCGAACAGGCTTTGGCTCCGGGACTAGGTGAAACCGTCCCGGA

No hit to direct repeat database and spacer database.

>5-SewaE-CRISPR_2
GATTGTTCTTTGAAAAGTTATTGATAAATGTTCATCTCATCATGGGTTCGCGGAAACGTGAAA
ATAAACCCGTGATTATCAAGGCCTTAAGTGGCCTACCGTTGCGCCCCGCAGGGGCGCATGAA
TTGAAACCTGTTTTGGGCGATGCTTGGGGCATTCAACCGGAGTTGCGCCCCGCAGGGGCGCAT
GAATTGAAACACAAACGCGCCGTATGTCGCATCTGCTTTCCACGTTGCGCCCCGCAGGGGCGC
ATGAATTGAAACACAGGCAATACAGACAAGCCGGTGAAGCACAAGAAGTTGCGCCCCGCAG
GGGCGCATGAATTGAAACGAAAAACGCCGGATATACATCTCTAAAAAGACAGTTGCGCCCCG
CAGGGGCGCATGAATTGAAACAGGTTCTCCGGGGTTGGGAACGTCAGATGTGCGAGTTGCGC
CCCGCAGGAGCGCATGAATTGAAACTGTGTTACAAATTCCCAAATCCCTTTAAGATCCTGTTG
CGCCCCGCAGGGGGCGCATGAATTGAAACAGGCAAGCGCAATTCAATTGGCAGAAGCCTACT
TGAAGGCAAAATGGAAATGGAAGGATTAAGATGAACAATGTTCAAATTTTGTGAACACCACC
TGTTC

Direct repeats 1 - 6:
CAS-Type I-C with 93% identity to CRISPR locus CP015249_6
Direct repeat 7:
CAS-Type I-C with 93% identity to CRISPR locus CP015249_4

No hit for spacers.

>6-SewaF-CRISPR_1
GAACATCTTATCACAGTGGACACATTCACCGCCACCAACCCGGTGGCCCCTCGCAAAGCCAG
GACGACACTTCTGATTTACAATCAGTTACAGAGATACTCTAGCAGTACCCAAGTAAATTTCAA
GGTAATGCGAGCAAACGGATGCTGGCCTATTCCAGCGTCGAGCACATGCTAGCAGTACCCAA
GTAAATTTCAAGGTAATGCGAGTCCCTGGATTATTGCGAGCCGTCGATGCCCCAGAAACTAGC
AGTACCCAAGTAAATTTCAAGGTAATGCGAGGTATAAATGGGAGACGGATAAAATTATCGAA
AAGGTGCTAGCAGTGCCCAAGTAAATTTCAAGGTAATGCGAGAAGATGTCTCTCGCAGGTTTT
ATAAGAAACGGGACACTAGCAGTACCCAAGTAAATTTCAAGGTAATGCGGGCAGCACCTGG
CTGAGCACCGTGCGACTGGGAAGAACTATTTATTACTATGAATAAATTTCAAAATGCAATAGA
TATAATTAAACAATTAACCATTAAACA

No hit to direct repeat database and spacer database.

>6-SewaF-CRISPR_2
CCGGTGGCGGCATCTTTTAAAGTTACGTAGGAAGGTTCCGGTGGTGTACCTAGCCGGTTAACT
TTTCCATAATAATGTGCTAATTTTTTCATCTTTCTCATACACTTCACTTTTTAGTTCGTTTGGGCG
TTGCAAGATGCAGTTTTTAACACCACACGAGTACGTCCTGTTGGACTCACATCACCTTTTAGTT
CGTTTGGGCGTTGCGAGCAGCGTATGTTAGTCATGGCGGCACGCCATATAGCGGCTCACATCA
CCTTTTAGTTCGTTTGGGCGTTGCGAGGGGTTGTAACTGATACACCACCATTGGCAATGCTGTC
ACTCACATCACCTTTTAGTTCGTTTGGGCGTTGCGAGAGCTTCTCTGTAACGTCCTGTATTGCA
ATGCCGTCGTCCTGTCTTTGCGAGGGGCCACCGGATTGGTGGCGGTGAATGTGTCCACTGTGA
TAAGATGTTT

No hit to direct repeat database and spacer database.

>6-SewaF-CRISPR_3
CATGATAAAGAAGAATTTGAAGATTATCCGGTAGATGCAATGTAAGAGTTGAAATATTAAAA
TGGGAGTTGACATTTGTGAAAAGTCTGATATAATGAGAGGCGAAAATTGAAATGCCAAAGAA
AAATTGAAAAAAAAAAATCGAAAAAGAGTTGACATTGATTAAAAGTCTGGTATAATGGTTGG
CGAAAATTGAAATACCAAAGAAAATTGGTAAAAAACAAAAATTGAACACTATTTATCTGGTA
GTATGAGAAATCGATTACAAAAACGGAATAGAGCCGAGGGCGATTGGACGCTCAATAGTAA
G

No hit to direct repeat database and spacer database.

References

1. Grissa, I.; Vergnaud, G.; Pourcel, C. CRISPRFinder: A Web Tool to Identify Clustered Regularly Interspaced Short Palindromic Repeats. *Nucleic Acids Res* **2007**, *35*, W52-57, doi:10.1093/nar/gkm360.
2. Couvin, D.; Bernheim, A.; Toffano-Nioche, C.; Touchon, M.; Michalik, J.; Néron, B.; Rocha, E.P.C.; Vergnaud, G.; Gautheret, D.; Pourcel, C. CRISPRCasFinder, an Update of CRISRFinder, Includes a Portable Version, Enhanced Performance and Integrates Search for Cas Proteins. *Nucleic Acids Res* **2018**, *46*, W246–W251, doi:10.1093/nar/gky425.
